# Mechanistic biomarkers for cancer vaccine development: from cancer cell biology to neoantigen-guided precision immunotherapy

**DOI:** 10.3389/fcell.2026.1897032

**Published:** 2026-08-21

**Authors:** Dario Rusciano

**Affiliations:** Independent Researcher, Livorno, Italy

**Keywords:** antigen presentation, artificial intelligence, cancer vaccines, colorectal cancer, HLA, immunopeptidomics, mechanistic biomarkers, neoantigens

## Abstract

Cancer vaccines have resurged in oncology because they address a central question in precision medicine: whether the molecular identity of a tumor can be converted into an immune target that is both specific and clinically useful. Preventive vaccines against oncogenic viruses have already shown that immune intervention can reduce the burden of virus-associated cancers. Therapeutic cancer vaccines face a more difficult task, because established tumors arise from self-tissues, change over time, and often acquire mechanisms that limit antigen presentation, T-cell entry, or immune-mediated killing. This review examines cancer vaccines as biomarker-driven tools within precision oncology. The focus is not only on vaccine platforms, but on the biological requirements that make an antigen suitable for therapeutic targeting. Tumor-specific mutations, viral antigens, recurrent driver alterations, frameshift peptides, cancer-testis antigens, and personalized neoantigens may all provide vaccine targets, but their presence alone is not enough. A clinically relevant vaccine antigen should be expressed by tumor cells, processed and presented through HLA molecules, recognized by functional T cells, and sufficiently retained during tumor evolution. This distinction is particularly important because sequencing and computational prediction now generate many candidate neoantigens whose immunological relevance still requires experimental confirmation. Particular attention is given to antigen-presentation defects, clonal and subclonal heterogeneity, tumor microenvironment barriers, circulating tumor DNA-defined minimal residual disease, and immune-response monitoring. Colorectal cancer is used as a working model because microsatellite instability-high/mismatch repair-deficient tumors, microsatellite-stable tumors with immune-resistant features, and recurrent alterations in MMR genes, KRAS, BRAF, adenomatous polyposis coli, and TP53 illustrate how cancer-cell signaling, neoantigen generation, tumor microenvironment remodeling, and patient stratification intersect. Overall, cancer vaccines are unlikely to become universal stand-alone treatments for advanced solid tumors. Their most credible role may emerge in molecularly selected patients, adjuvant therapy, minimal residual disease, virus-associated malignancies, and rational combinations with checkpoint inhibitors or tumor microenvironment-modulating agents.

## 1 Introduction: cancer vaccines as a model of mechanistic precision oncology

Cancer vaccines are making a comeback at a time when tumors are no longer viewed only by tissue of origin, but also by their mutational profile, immune environment, evolutionary history, and likelihood of responding to treatment. For many years, therapeutic cancer vaccination was attractive in principle but disappointing in practice. The problem was not that tumors are always invisible to the immune system. Rather, established cancers originate from self-tissues and often survive by losing antigens, reducing antigen presentation, excluding effector cells, exploiting metabolic stress, or exhausting T cells (Hanahan, 2022; Schreiber et al., 2011).

During life, cells with potentially oncogenic alterations probably arise more often than clinically evident cancers would suggest. Many are eliminated or held in check by immune surveillance before they become detectable. Clinically relevant tumors can therefore be viewed, at least in part, as lesions that have survived immune pressure, entered immune equilibrium, or acquired mechanisms of escape. This idea, usually described as cancer immunoediting, helps explain why established tumors are difficult vaccine targets: at diagnosis, they may already have selected features that reduce antigen visibility or suppress immune function (Schreiber et al., 2011).

Preventive vaccination provides a useful contrast. Vaccines against hepatitis B virus and human papillomavirus target foreign viral antigens before malignant transformation and have reduced the incidence of virus-associated cancers (Chang et al., 2009; Arbyn et al., 2018). Therapeutic cancer vaccines must work after a tumor has developed, often when heterogeneity and immune suppression are already present. Modern vaccine development has therefore moved away from simply administering tumor antigens and hoping for protection. It now asks which antigens are visible to the immune system, which patients are most likely to respond, and which clinical setting offers the best chance for immune control.

This contrast also explains why many earlier therapeutic programs disappointed. Vaccination against shared self-antigens often produced measurable circulating immune responses without objective tumor regression; an influential analysis reported an objective response rate of 2.6% among 440 patients treated in National Cancer Institute vaccine trials (Rosenberg et al., 2004). Tolerance to self-antigens, weak or short-lived effector function, bulky late-stage disease, antigen heterogeneity or loss, and a suppressive tumor microenvironment all contributed. Sipuleucel-T later showed that a vaccine-like cellular product could prolong overall survival in metastatic castration-resistant prostate cancer despite no improvement in time to objective disease progression (Kantoff et al., 2010). This divergence exposed a second difficulty: radiographic response criteria may miss delayed benefit, but immunogenicity alone cannot establish tumor control. Current neoantigen strategies should be judged against both lessons.

Within precision oncology, a useful vaccine target is not defined only by mutation, overexpression, or computational prediction. It should connect a cancer-cell alteration with a functional immune mechanism: expression in tumor cells, processing by antigen-presentation machinery, display by HLA molecules, recognition by tumor-reactive T cells, and sufficient persistence during tumor evolution (Schumacher and Schreiber, 2015; Yarchoan et al., 2017). Each step may fail. This is why antigen discovery and antigen validation are not the same thing. Sequencing can identify many candidates, but some are not transcribed, not translated, not presented, or not found in the tumor cells that matter clinically (Bassani-Sternberg et al., 2015; McGranahan et al., 2017).

Neoantigens remain central because they offer a level of tumor specificity that many self-antigens cannot provide. Early melanoma studies showed that personalized peptide or RNA vaccines could be designed from each patient’s tumor mutational profile and induce neoantigen-specific T-cell responses (Ott et al., 2017; Sahin et al., 2017). Later work showed persistent immune memory and epitope spreading (Hu et al., 2021). More recent individualized mRNA-based vaccines in resected melanoma and pancreatic cancer suggest that vaccination may be most useful when tumor burden is low, immune suppression is not yet dominant, or checkpoint blockade helps maintain vaccine-induced T-cell activity (Weber et al., 2024; Rojas et al., 2023).

Colorectal cancer illustrates the opportunities and limits of this approach. Microsatellite instability-high and mismatch repair-deficient tumors often accumulate frameshift mutations and respond better to checkpoint blockade than microsatellite-stable tumors (Le et al., 2017; Llosa et al., 2015). Recurrent alterations in KRAS, BRAF, APC, TP53, and mismatch repair genes can also influence antigen generation, immune escape, and target retention (Yoon et al., 2025). The subsequent sections use this disease as a working model for connecting molecular alterations to therapeutic choice.

### 1.1 Literature search and selection criteria

This review was developed as a narrative and mechanistic synthesis of the literature rather than as a systematic review or meta-analysis. Relevant publications were identified mainly through PubMed, using combinations of terms including “cancer vaccines,” “neoantigens,” “tumor antigens,” “personalized cancer vaccine,” “shared neoantigens,” “HLA presentation,” “immunopeptidomics,” “tumor microenvironment,” “immune checkpoint blockade,” “cancer immunoediting,” “colorectal cancer,” “KRAS,” “BRAF,” “APC,” “TP53,” “mismatch repair deficiency,” “microsatellite instability,” “artificial intelligence,” and “cancer immunotherapy biomarkers.” Additional articles were selected from the reference lists of relevant reviews and primary studies.

Priority was given to peer-reviewed articles that provided mechanistic, translational, or clinical insight into antigen generation, antigen presentation, tumor immune escape, vaccine target selection, functional validation, and biomarker-guided patient stratification. Both original studies and authoritative reviews were considered, particularly when they helped clarify how cancer-cell biology can be connected to vaccine-relevant biomarkers.

Publications were selected for relevance to the conceptual framework of the review rather than by a formal quantitative screening procedure. No meta-analysis was performed.

## 2 From cancer-cell alterations to vaccine-relevant antigens

The first step in designing a cancer vaccine is not the platform, but the target. A tumor antigen must be different enough from normal tissues to be safely attacked, expressed enough to be seen, and stable enough to remain present while the immune response develops. It also has to enter the ordinary biology of antigen processing and presentation, becoming a peptide-HLA complex that T cells can recognize within a tumor environment where they can still function (Saxena et al., 2021; Lin et al., 2022).

Malignant cells acquire their antigenic identity through processes that also drive cancer progression: mutation, genomic instability, viral transformation, altered transcription, abnormal splicing, epigenetic deregulation, lineage infidelity, and selection under immune pressure. Some changes generate antigens shared across patients; others create private targets unique to one tumor. Some are tumor-specific, whereas others are tumor-associated antigens (TAA) that are overexpressed or aberrantly expressed but not fully restricted to malignant tissue (Buona et al., 2020; Coulie et al., 2014). This distinction matters because it affects immune tolerance, safety, and the strength of the vaccine response.

Historically, many vaccines targeted TAA such as differentiation antigens, cancer-testis antigens, or overexpressed proteins. These targets are useful because they can support off-the-shelf approaches, but they often face partial immune tolerance and, in some cases, the risk of off-tumor toxicity (Buona et al., 2020; Coulie et al., 2014). Neoantigens offered a more tumor-specific logic. A mutation that changes a protein sequence may create a peptide not encountered during thymic selection; if processed, presented, and recognized, it can become a selective immune target (Schumacher and Schreiber, 2015; Yarchoan et al., 2017; Saxena et al., 2021). Yet only a small fraction of somatic mutations reaches this point.

These antigen classes solve different problems and introduce different forms of uncertainty. Table 1 summarizes their main translational advantages and limitations.

**TABLE 1:** Major classes of vaccine antigens and their principal translational trade-offs.

Antigen class	Representative examples	Main advantages	Main limitations
Viral antigens	HPV E6/E7; HBV antigens	Foreign, often conserved, and less constrained by self-tolerance; compatible with shared products	Limited to virus-associated tumors; established cancers may retain strong presentation and microenvironmental escape mechanisms
Tumor-associated antigens	Cancer-testis, differentiation, or overexpressed antigens (e.g., NY-eso-1, MAGE-A, gp100, HER2)	Broad applicability and off-the-shelf manufacturing	Central or peripheral tolerance; variable tumor restriction; off-tumor toxicity and antigen-loss risk
Shared neoantigens	KRAS, BRAF, or TP53 hotspots; recurrent mismatch-repair frameshifts	Tumor specificity with stratified, reusable manufacturing	Restricted by mutation prevalence, HLA genotype, processing, clonality, and retention
Personalized neoantigens	Private substitutions, indels, frameshifts, and fusions	Patient-specific tumor targeting and multivalent design	Requires tissue, sequencing, computation, rapid manufacture, and quality control; predictions remain uncertain and targets may evolve
Noncanonical and fusion-derived tumor-specific antigens	Alternative reading frames, abnormal splicing, noncoding translation, endogenous retroviral elements, and fusion junctions	Expands the target space and may reveal highly tumor-restricted peptides	Annotation and detection are less standardized; low abundance is common; natural presentation and immunogenicity require direct validation

Frameshift peptides are particularly relevant in mismatch repair-deficient tumors. Microsatellite instability can generate insertion or deletion errors in coding sequences, creating abnormal peptides that may be more foreign than single amino-acid substitutions. This makes them attractive vaccine candidates, but they still require the same chain of validation: expression, processing, HLA presentation, T-cell recognition, and persistence in tumor cells (Yarchoan et al., 2017; Saxena et al., 2021). Viral antigens provide a cleaner case because they are foreign and linked to oncogenic transformation, as in HPV-associated or HBV-associated cancers (Chang et al., 2009; Arbyn et al., 2018). While therapeutic vaccination against virus-associated tumors is still difficult, the target it aims for is less restricted by self-tolerance than many TAA.

The antigenic landscape of TAA is broader than the classical protein-coding exome. Noncoding regions, aberrantly translated transcripts, alternative reading frames, endogenous retroviral elements, and cancer-specific splicing or epigenetic events can also generate tumor-specific antigens (Apavaloaei et al., 2020; Laumont et al., 2018). These unconventional sources make the target space larger, but they also increase the need for proof that the peptide is truly produced and presented. Mass spectrometry-based immunopeptidomics can directly identify peptides bound to HLA molecules, while functional T-cell assays test whether those peptides are immunogenic (Becker et al., 2022). Fusion proteins add a related example: their junctional sequences may be unique to the tumor and shared among patients with the same fusion, but only if they are processed, presented, and recognized do they become useful vaccine targets (Yang et al., 2019).

**FIGURE 1:**
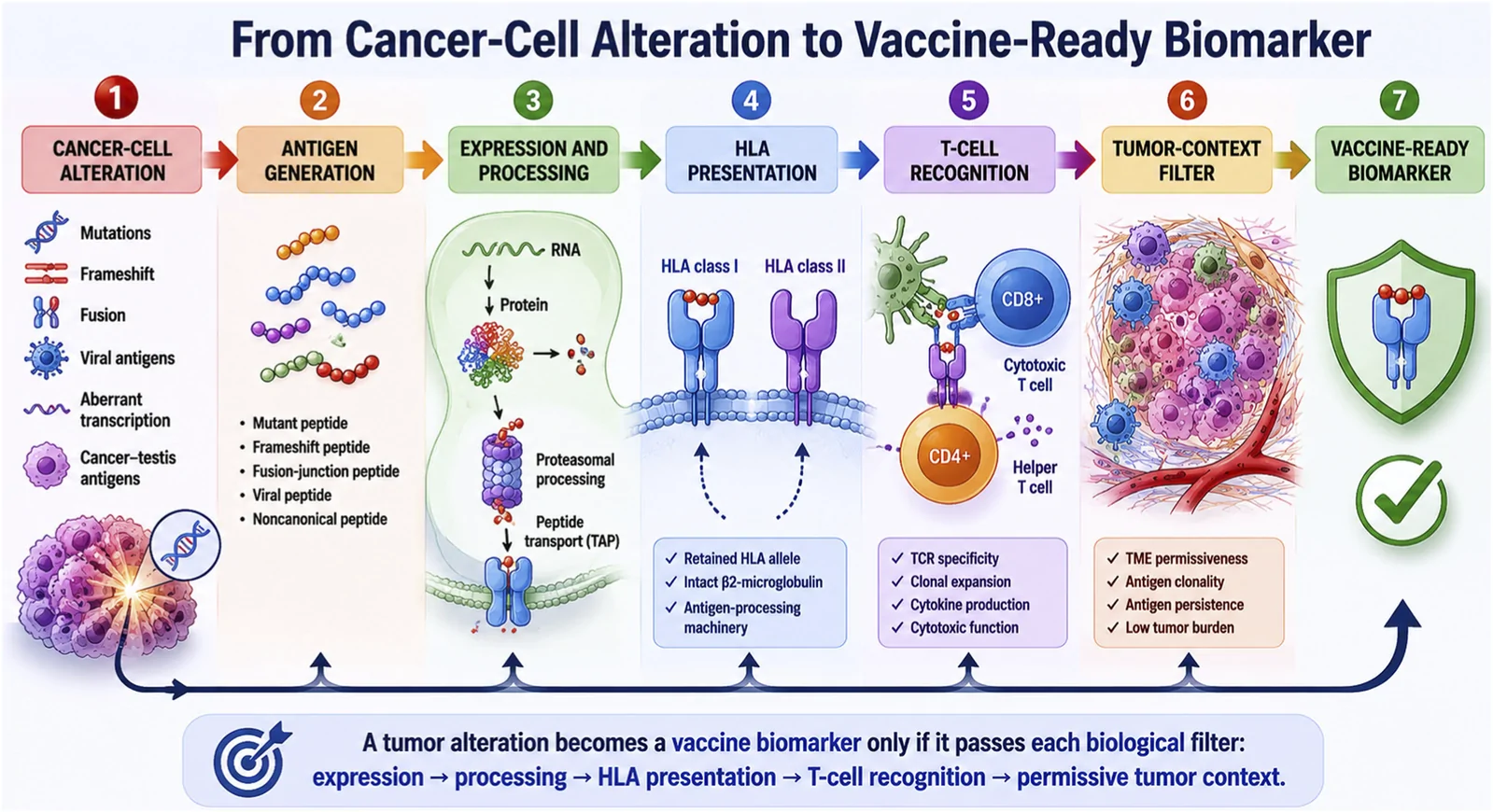
From cancer-cell alteration to vaccine-ready biomarker. A tumor alteration becomes clinically relevant for vaccination only after passing several biological filters. Mutations, frameshifts, gene fusions, viral antigens, aberrant transcriptional programs, or cancer-testis antigens may generate candidate peptides, but these must be expressed, processed, presented by HLA class I or II molecules, and recognized by functional CD8^+^ or CD4^+^ T cells. Therapeutic value also depends on HLA retention, antigen clonality and persistence, microenvironmental permissiveness, and tumor burden. Antigen detection is therefore the beginning of validation, not its endpoint. This conceptual figure was generated using ChatGPT (ChatGPT 5.5 Thinking) from author-defined scientific content and was subsequently reviewed, corrected, and finalized using GIMP 2.10.30.

Some driver alterations create vaccine relevance indirectly. Loss of adenomatous polyposis coli (APC) function in colorectal cancer may not yield a simple mutant peptide target, but dysregulated Wnt/beta-catenin signaling can reshape stemness programs, immune exclusion, and downstream antigenic states (Yoon et al., 2025). KRAS, BRAF, and TP53 may similarly matter both as potential sources of mutant peptides and as signaling events that alter immune recognition. For this reason, a vaccine biomarker should be judged across several layers: tumor specificity, biological relevance, immune visibility, functional recognition, and clinical context. Computational tools can help organize genomic, transcriptomic, and HLA-binding information for antigen prioritization, but they remain primarily tools for prioritization (Zhou et al., 2021). This stepwise transition from cancer-cell alteration to vaccine-ready biomarker is summarized in Figure 1.

## 3 Neoantigens as mechanistic therapeutic biomarkers

Neoantigens connect the genetic individuality of a tumor to a potentially selective immune target. They can arise from single-nucleotide variants, insertions or deletions, frameshifts, gene fusions, or other tumor-restricted events. When the altered peptide is absent from normal tissues, vaccination may focus immunity on malignant cells while limiting damage to healthy tissue (Schumacher and Schreiber, 2015; Yarchoan et al., 2017).

Mutation detection nevertheless requires several exclusions before a candidate can be called a functional neoantigen. The variant may not be transcribed; the protein may not yield the predicted peptide; HLA binding may be weak; or expression may be confined to a minor subclone (Bassani-Sternberg et al., 2015; Becker et al., 2022). Even confirmed T-cell recognition does not guarantee rejection when lymphocytes are exhausted, spatially excluded, or locally suppressed. Here, the term mechanistic biomarker is reserved for an antigen whose validation helps explain the likelihood of response, a defined mode of failure, or the rationale for a combination treatment (Schumacher and Schreiber, 2015; Yarchoan et al., 2017; Becker et al., 2022).

Personalized neoantigens are derived from the patient-specific mutational profile of an individual tumor. The usual workflow involves sequencing tumor and normal DNA, identifying somatic mutations, typing HLA, and prioritizing candidates according to predicted binding, expression, clonality, and other features. This strategy was shown to be feasible in melanoma, where personalized peptide and RNA vaccines induced neoantigen-specific T-cell responses and, in some patients, persistent immune memory (Ott et al., 2017; Sahin et al., 2017; Hu et al., 2021). Its strength is precision; its limitation is that production is complex, time-sensitive, and dependent on imperfect prediction. The approach is most convincing in adjuvant or minimal residual disease settings, where fewer malignant cells need to be eliminated and immune suppression may be less dominant (Weber et al., 2024; Rojas et al., 2023).

Shared neoantigens follow a different logic. They arise from recurrent mutations or recurrent abnormal sequences in biologically defined patient groups, including KRAS mutations, BRAF V600E, selected TP53 hotspot mutations, and frameshift peptides in mismatch repair-deficient tumors (Yoon et al., 2025). They support a stratified, rather than fully individualized, form of precision medicine. A patient with a defined mutation and a compatible HLA type may receive a vaccine directed against a recurrent mutant peptide, reducing manufacturing complexity while preserving molecular selection.

KRAS illustrates both the attraction and the difficulty of shared neoantigens. G12D and G12V mutations are frequent and can generate mutant peptides absent from normal tissues. Clinical and experimental work has shown that mutant KRAS can be recognized by T cells in selected HLA contexts (Tran et al., 2016). However, the same KRAS mutation does not create the same immune target in every patient; presentation depends on HLA genotype, processing, peptide binding, and tumor-cell presentation capacity. Frameshift peptides in mismatch repair-deficient tumors are another attractive shared-antigen setting and have opened therapeutic and interception-oriented vaccine strategies, including in Lynch syndrome (Kloor et al., 2020).

Clonality is central. A clonal neoantigen is present in most tumor cells because it arose early; a subclonal neoantigen appears later and is found only in part of the tumor. Neoantigens shared by most malignant cells are more likely to support effective antitumor immunity than those limited to smaller subclones (McGranahan et al., 2016). A vaccine against a subclonal target may eliminate only antigen-positive cells and leave antigen-negative populations behind. This is why multivalent vaccines are appealing, although the quality of selected antigens remains more important than their number.

Neoantigen quality is more important than mutation number alone. Tumor mutational burden is only an approximate indicator of how many useful immune targets a tumor may contain. Expression level, peptide-HLA stability, antigen processing, clonal distribution, and the degree of difference from the normal peptide all influence immunogenicity. Models that combine these features may predict immune responsiveness better than mutation count alone (Luksza et al., 2017). In pancreatic cancer, long-term survival has been linked not simply to more neoantigens, but to neoantigens with favorable qualitative properties (Balachandran et al., 2017).

Vaccines and checkpoint inhibitors exploit the same antigen-specific biology at different points. Vaccination seeks to prime or expand selected tumor-reactive clones; CTLA-4 or PD-1/PD-L1 blockade can release inhibitory constraints on clones that are already present or newly induced (Pardoll, 2012; Gubin et al., 2014). Combining the two is therefore rational only when both a credible peptide-HLA target and a reversible checkpoint-related barrier are demonstrated. Candidate confidence can be graded from predicted, to expressed, naturally presented, T-cell recognized, and finally actionable; the last category also requires adequate clonality, tumor retention, and a feasible clinical setting (Schumacher and Schreiber, 2015; Yarchoan et al., 2017; Becker et al., 2022). This hierarchy avoids equating mutation burden or algorithmic rank with therapeutic value.

## 4 Antigen presentation: The functional bottleneck of cancer vaccines

A cancer vaccine can work only if the immune system can see what the vaccine is trying to target. Tumor sequencing may identify a mutation, and prediction tools may suggest HLA binding, but the decisive question is whether the peptide is actually displayed on the tumor-cell surface. If not, even an attractive antigen remains invisible.

Antigen presentation lies between antigen discovery and immune killing. The altered protein must be degraded, transported, loaded onto HLA molecules, and carried to the surface. Only then can CD8^+^ T cells detect the peptide-HLA complex. Defects in this pathway are common in cancer and are major routes of immune escape (Dhatchinamoorthy et al., 2021; Maggs et al., 2021). This is why vaccine development has moved from simple HLA-binding prediction toward approaches that also consider RNA expression, protein abundance, antigen processing, immunopeptidomics, and functional T-cell testing.

HLA class I molecules present intracellular peptides to CD8^+^ cytotoxic T cells. Tumor cells may downregulate HLA class I, alter beta-2-microglobulin, impair transporter associated with antigen processing function, change proteasomal processing, or suppress transcriptional regulators of antigen-presentation genes. Some defects are genetic and difficult to reverse; others are epigenetic, transcriptional, or cytokine-dependent and may be more dynamic (Maggs et al., 2021; Garrido et al., 2016). Loss of beta-2-microglobulin is a clear hard barrier: without it, HLA class I molecules cannot be stabilized at the cell surface, and vaccine-induced CD8^+^ T cells may expand without recognizing malignant cells (Zaretsky et al., 2016; Gettinger et al., 2017).

HLA loss of heterozygosity is subtler. A tumor may retain some HLA expression but lose the allele that presents the most immunogenic neoantigens. Studies in lung cancer have shown that allele-specific HLA loss can occur during tumor evolution and contribute to immune escape (McGranahan et al., 2017). Interferon signaling is also important. Interferongamma can strengthen antigen-presentation machinery, but defects in pathways such as JAK1/JAK2 signaling can reduce this response and have been linked to resistance to PD-1 blockade (Zaretsky et al., 2016). NLRC5, a major regulator of MHC class I-related genes, further illustrates how tumor visibility can be shaped by broader transcriptional programs (Yoshihama et al., 2016).

HLA class II should not be ignored. CD4^+^ T cells can support dendritic-cell activation, sustain CD8^+^ responses, produce cytokines, and contribute to immune memory. Long peptides, RNA vaccines, dendritic-cell vaccines, and other multivalent platforms may therefore benefit from inducing both CD4^+^ and CD8^+^ responses (Saxena et al., 2021; Lin et al., 2022). Antigen presentation is not only a tumor-cell property; dendritic cells and other professional antigen-presenting cells are needed to prime T cells and shape response quality.

For therapeutic biomarkers, the consequence is practical. A tumor containing a predicted neoantigen but lacking the relevant HLA allele, beta-2-microglobulin, or key processing machinery is unlikely to respond to a vaccine relying mainly on CD8^+^ recognition of that peptide. Conversely, a tumor with preserved HLA expression, evidence of antigen processing, and inflammatory responsiveness may be more credible, especially if the antigen is clonal and tumor burden is limited. Some presentation defects may be partial or reversible, making combination therapy reasonable, but the purpose should be to correct a defined barrier rather than simply add treatments.

## 5 Signature mutations as dual biomarkers: oncogenic drivers and vaccine targets

Some tumor alterations are important because they drive cancer growth; others are important because they create antigens. Often, these categories overlap. A driver mutation may shape proliferation, invasion, drug resistance, antigenicity, immune escape, and sensitivity to immune-based treatments. If it is both biologically important and immunologically visible, it can become more than a molecular label. It can become a therapeutic biomarker.

These dual roles are particularly visible in colorectal cancer. Mismatch repair deficiency can create recurrent frameshift peptides, whereas KRAS, BRAF, APC, and TP53 alterations either provide mutant epitopes or remodel presentation, immune infiltration, and escape (Yoon et al., 2025). The relevant question is not simply which mutation is present, but what antigenic and immune state it produces.

Defective mismatch repair changes the antigenic landscape through replication errors in coding microsatellites, some of which generate recurrent frameshift peptides (Kloor et al., 2020; Willis et al., 2020). A high frameshift load contributes to immunogenicity, but outcome still depends on HLA presentation and counter-inhibitory pathways (Le et al., 2017; Llosa et al., 2015; Willis et al., 2020). In established mismatch repair-deficient cancers, a frameshift-peptide vaccine was safe and immunogenic; in a Lynch syndrome mouse model, vaccination reduced tumor burden and improved survival (Kloor et al., 2020; Gebert et al., 2021). These findings support both therapeutic and interception-oriented development without implying that microsatellite instability-high status alone predicts vaccine efficacy.

KRAS is a strong example of a shared driver target. Mutations such as G12D, G12V, and G12C are frequent in colorectal, pancreatic, and lung cancers. They activate pathways that support tumor growth, and mutant KRAS peptides can be recognized by T cells in selected HLA contexts (Tran et al., 2016; Lu et al., 2023). The limitation is that a shared mutation does not automatically create a shared immune target for every patient. Presentation depends on HLA genotype, processing, peptide binding, and the tumor’s presentation capacity. KRAS also matters beyond the peptide, because KRAS-driven signaling can influence cytokines, myeloid recruitment, stromal interactions, and other resistance features.

BRAF V600E is another recurrent driver with context-dependent immunological relevance. In melanoma, BRAF V600E-specific CD4^+^ T cells have been associated with a complete clinical response after adoptive transfer of tumor-infiltrating lymphocytes (Veatch et al., 2018). In colorectal cancer, BRAF V600E is linked to distinct molecular and clinical features, including association with microsatellite instability in some tumors and aggressive behavior in others (Yoon et al., 2025; Fanelli et al., 2020). A BRAF-mutant MSI-H tumor and a BRAF-mutant microsatellite-stable tumor may not be equally suitable for vaccination, even if the driver mutation is identical.

APC loss shows the indirect side of vaccine biology. In colorectal cancer, APC loss deregulates Wnt/beta-catenin signaling and supports proliferation and stemness, but it does not usually provide a simple shared mutant peptide target (Yoon et al., 2025). Wnt/beta-catenin activation has been linked to immune exclusion and poor T-cell infiltration (Spranger et al., 2015; Luke et al., 2019). A vaccine may induce antigen-specific T cells, yet those cells may not reach tumor nests. At the same time, downstream stemness-associated programs may create secondary antigenic opportunities. The KISIMAAscl2 model illustrates this principle. Ascl2 is a transcription factor associated with intestinal stem-cell programs and Wnt-driven tumorigenesis. In a mouse model of sporadic colon adenomas, vaccination against Ascl2 reduced adenoma formation, suggesting that vaccine targets may also arise from the biological state created by oncogenic pathway deregulation, rather than only from the mutated driver itself (Belnoue et al., 2021). This does not yet establish Ascl2 as a clinically validated colorectal cancer vaccine target, but it supports the broader idea that pathwaydependent tumor programs can generate indirect vaccine opportunities.

**FIGURE 2:**
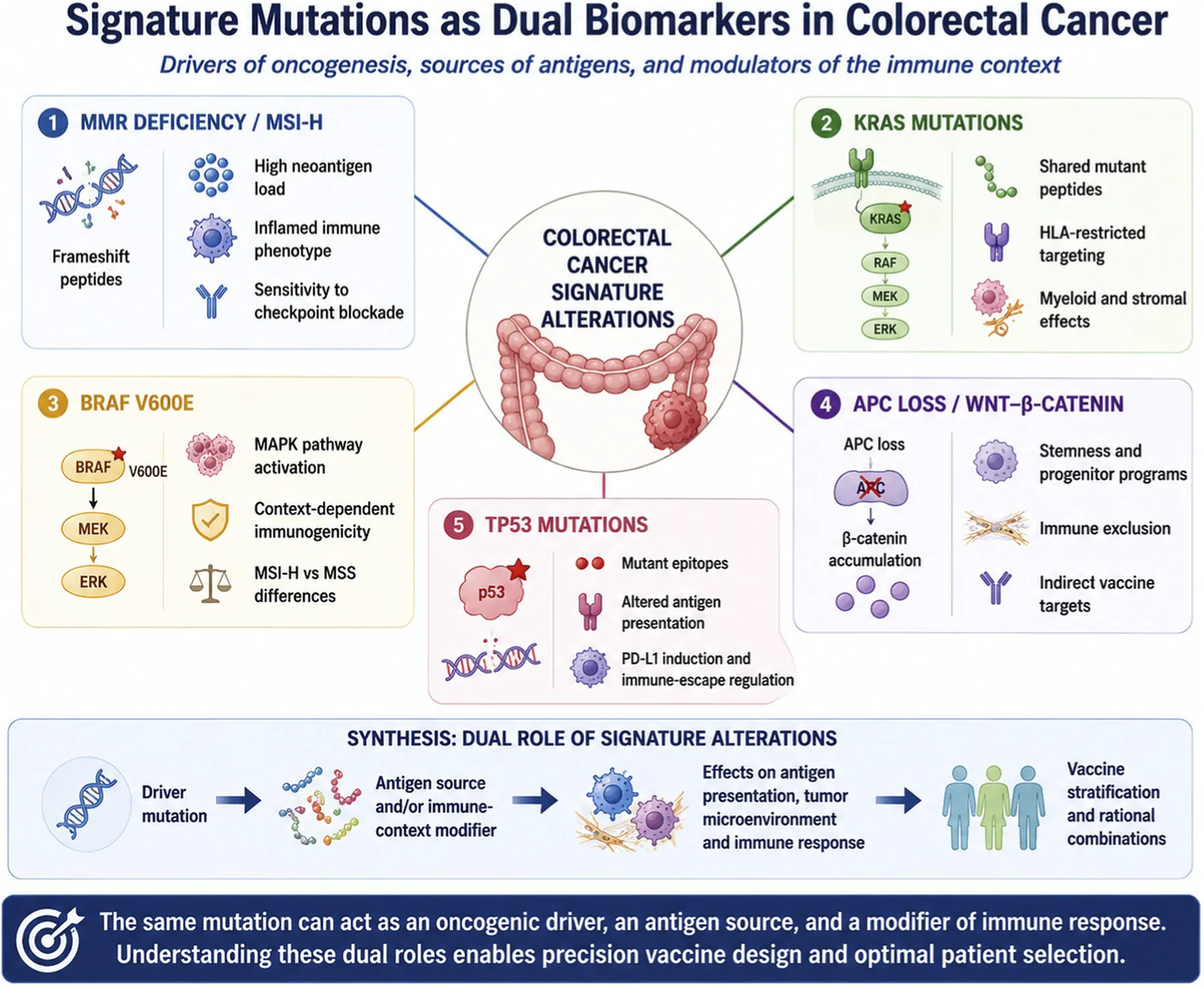
Signature mutations as dual biomarkers in colorectal cancer. Mismatch repair deficiency/microsatellite instability-high status can generate frameshift peptides and an inflamed immune phenotype; KRAS and BRAF mutations may act as oncogenic drivers and shared neoantigen sources; APC loss can promote Wnt/β-catenin-driven stemness and immune exclusion; and TP53 mutations may generate mutant epitopes while also influencing antigen presentation and immune escape. The same alteration can therefore inform antigen selection, patient stratification, and the rationale for combination therapy. This conceptual figure was generated using ChatGPT (ChatGPT 5.5 Thinking) from author-defined scientific content and was subsequently reviewed, corrected, and finalized using GIMP 2.10.30.

TP53 adds another layer of complexity to this discussion. Because TP53 is frequently altered across human cancers, recurrent hotspot mutations may in principle provide shared vaccine targets. Indeed, some TP53 hotspot mutations generate peptides recognized by T cells, and a common p53 mutation-derived peptide–HLA complex can be targeted with high specificity (Malekzadeh et al., 2019; Hsiue et al., 2021). Yet TP53 alterations also affect apoptosis, genomic stability, metabolism, antigen presentation, inflammatory signaling, PD-L1 expression, and interactions with the immune microenvironment (Wang et al., 2024). TP53 should therefore be treated both as a possible source of mutant epitopes and as a regulator of immune escape. The general meaning is that signature mutations should not be treated only as labels. Their value depends on clonality, antigen generation, HLA retention, tumor context, and clinical timing. The dual role of colorectal cancer signature alterations as oncogenic drivers, antigen sources, and immune-context modifiers is summarized in Figure 2.

## 6 Tumor microenvironment, heterogeneity, and evolution

A vaccine can generate antigen-specific T cells, but those cells must still work inside a living tumor. The tumor microenvironment is not a passive background. It includes immune cells, fibroblasts, endothelial cells, extracellular matrix, soluble mediators, and metabolic gradients. Some tumors allow T cells to enter and kill; others keep them outside or silence them after entry. For vaccines, the local environment determines whether immune recognition can become tumor control (Binnewies et al., 2018).

Human cancers show different immune states that do not simply follow tissue of origin. Large immunogenomic analyses have identified immune subtypes characterized by different balances of lymphocytes, macrophages, interferon signaling, transforming growth factor beta (TGF-β) activity, wound-healing programs, and checkpoint expression (Thorsson et al., 2018). In practical terms, tumors are often described as “hot” when they contain an active immune infiltrate and “cold” when they show little effector T-cell activity. This distinction is useful, but too simple. A more informative view separates immune-inflamed tumors, immune-excluded tumors, and immune-desert tumors (Binnewies et al., 2018; Thorsson et al., 2018; Chen and Mellman, 2017). This matters for vaccination because each pattern suggests a different barrier: insufficient priming, poor T-cell entry, stromal exclusion, or local immune suppression. In colorectal cancer, the type, density, and location of immune cells predict clinical outcome, showing that immune geography can be as important as immune abundance (Galon et al., 2006).

Checkpoint blockade has reinforced this idea. In melanoma, response to PD-1 blockade was associated with preexisting CD8^+^ T cells at the invasive margin and evidence that these cells were restrained by the PD-1/PD-L1 axis (Tumeh et al., 2014). Vaccination is different, but the principle is relevant: a tumor containing restrained T cells may benefit from checkpoint blockade, whereas a tumor without T-cell infiltration may also need improved priming, recruitment, or local inflammation. Dendritic cells are central to this process because they connect antigen release to T-cell priming; expanding and activating CD103+ dendritic-cell progenitors improved responses to PD-L1 blockade and BRAF inhibition in preclinical models (Salmon et al., 2016).

A T-cell-only account is nevertheless incomplete. B cells can present antigen to CD4^+^ T cells, secrete cytokines and antibodies, and organize with T follicular helper cells and dendritic cells in tertiary lymphoid structures. In a protein-vaccine study using NY-ESO-1, antibody and CD4^+^ responses preceded CD8^+^ responses, and immune complexes enhanced cross-presentation *in vitro* (Valmori et al., 2007). In melanoma and renal cell carcinoma, B-cell-rich tertiary lymphoid structures were associated with favorable checkpoint-blockade responses and distinct T-cell states (Cabrita et al., 2020; Helmink et al., 2020). These observations do not make B-cell abundance a universal vaccine biomarker: some B-cell phenotypes are regulatory, and antibodies have more direct access to surface or extracellular antigens than to intracellular mutant peptides. Even so, antigen-specific antibodies, B-cell clonotypes, and tertiary lymphoid structure maturation merit inclusion in immune monitoring, especially for protein, viral-vector, and *in situ* platforms.

Myeloid and stromal cells often explain why vaccine-induced responses fail locally. Myeloid-derived suppressor cells and tumor-associated macrophages can reduce T-cell function through arginine depletion, nitric oxide and reactive oxygen species, immunosuppressive cytokines, checkpoint ligands, and regulatory T-cell support (Gabrilovich, 2017; Cassetta and Pollard, 2018). Cancer-associated fibroblasts and TGF-beta signaling can restrict T-cell access and promote immune exclusion. In urothelial cancer and colorectal cancer models, TGF-beta-related stromal programs were associated with T-cell exclusion and resistance to immunotherapy (Mariathasan et al., 2018; Tauriello et al., 2018). Abnormal vasculature and VEGF signaling can also limit lymphocyte trafficking and promote suppressive immune states; anti-angiogenic therapy may therefore support immunotherapy by remodeling the vascular and immune environment (Fukumura et al., 2018).

Metabolic conditions add another barrier. Hypoxia, nutrient depletion, lactate accumulation, adenosine signaling, and altered amino-acid metabolism can reduce effector T-cell activity (Vaupel and Multhoff, 2017). These features are not always easy to measure clinically, but they remind us that microenvironmental biomarkers should not be limited to cell counts. The functional state of the tissue matters as much as its cellular composition.

Spatial heterogeneity changes both antigen prevalence and immune exposure. Multi-region sequencing in renal and lung cancers demonstrated branched evolution with coexisting clonal and subclonal events (Gerlinger et al., 2012; Jamal-Hanjani et al., 2017). Early, trunk alterations are more likely to be shared across regions and metastases; later branch alterations may be confined to one lesion or cell population (McGranahan et al., 2016; Jamal-Hanjani et al., 2017). A single biopsy can therefore overestimate target coverage, while bulk variant allele frequency may be distorted by tumor purity and copy-number changes.

Distribution is not static. The local immune context applies site-specific selection: longitudinal analysis of colorectal cancer metastases found neoantigen depletion where proliferating tumor cells were spatially close to T cells, whereas immune-privileged sites preserved escape clones (Angelova et al., 2018). Lung cancers can likewise lose expressed neoantigens or the HLA alleles needed to present them during progression (McGranahan et al., 2017; Rosenthal et al., 2019). Checkpoint blockade and tumor-T-cell interactions add further selection, favoring antigen-loss or presentation-defective populations (Anagnostou et al., 2017; Verdegaal et al., 2016). Clonal status should therefore be treated as a spatiotemporal estimate, not a fixed property of the initial biopsy.

Vaccine design should preferentially cover trunk antigens detected across available regions or lesions and, when needed, combine targets carried by independent branches. Multi-region sampling is informative when feasible; serial circulating tumor DNA can then track selected variants, tumor burden, and emergent clones (Abbosh et al., 2017). It cannot reveal whether a peptide is still presented or whether immune architecture has changed. Re-biopsy and renewed HLA or immunopeptidomic assessment remain important at progression when the result could alter treatment.

## 7 Multi-omics, artificial intelligence, immunopeptidomics, and validation

Cancer vaccine development now uses information at a scale that was not available a generation ago. Tumor sequencing, RNA profiling, HLA typing, mass spectrometry, single-cell analysis, spatial methods, and computational modeling can all contribute to target selection. Vaccine design can now begin from the molecular profile of an individual tumor and move toward a short list of candidates suitable for vaccination (Hundal et al., 2020; Reynisson et al., 2020). More data, however, do not automatically create better targets. Computational and multi-omic approaches are tools for prioritization, not final proof that a peptide is naturally presented, immunogenic, or clinically useful.

The usual workflow starts with tumor and matched-normal sequencing. Whole-exome or whole-genome sequencing identifies somatic mutations, RNA sequencing estimates expression, and HLA typing predicts which peptides may bind the patient’s HLA molecules. pVACtools is one widely used workflow for neoantigen identification, prioritization, and visualization (Hundal et al., 2020). HLA-binding prediction is among the most established steps. NetMHCpan and NetMHCIIpan use machine-learning strategies trained on binding and mass spectrometryeluted ligand data (Reynisson et al., 2020), while MHCflurry 2.0 also incorporates features of antigen processing (O'Donnell et al., 2020). Binding matters, but it is only one part of presentation.

Artificial intelligence and machine learning may help integrate variables such as mutation type, peptide sequence, HLA binding, gene expression, variant allele frequency, clonality, processing probability, self-similarity, and immunopeptidomic information (Hundal et al., 2020; Cai et al., 2023). These tools can compare many candidates and detect patterns that are hard to capture with simple rules. They cannot guarantee that a ranked peptide will become an effective vaccine target. The same caution applies to T-cell receptor-peptide-MHC prediction. Recent deep-learning models have attempted to predict TCR-pMHC interaction from receptor, epitope, and MHC-related information (Zhang et al., 2023), but the complexity of T-cell repertoires and limited training data mean that functional assays remain necessary.

Immunopeptidomics approaches the problem from the opposite direction. Instead of asking which peptides might be presented, it identifies peptides actually bound to HLA molecules by mass spectrometry (Becker et al., 2022; Cai et al., 2025). This can strengthen prioritization because it shows that a candidate has passed through antigen processing and presentation. Recent pan-cancer work has shown that canonical and noncanonical peptides can both contribute to the presented antigen landscape (Cai et al., 2025). Still, immunopeptidomics requires enough tissue, careful preparation, sensitive mass spectrometry, and robust analysis. Low-abundance peptides may be missed, and detection alone does not prove immunogenicity.

Single-cell and spatial technologies add information about cellular state and location. They can show whether an antigen is expressed only in a minor tumor population, whether antigen-presentation programs are weak, or whether immune cells are spatially separated from antigen-expressing cells. These methods are already being used to study response and resistance to checkpoint inhibitors, and the same information is relevant to vaccine development (Liu et al., 2024). Proteogenomic approaches attempt to combine mutation, transcript, protein, HLA-binding, and immunopeptidomic data into a more complete view of credible antigens (Huber et al., 2025). Interactive tools such as pVACview can then support transparent expert review and selection (Xia et al., 2024).

For clinical translation, reproducibility matters. Different pipelines may rank the same tumor differently because they use different variant callers, transcript annotations, HLA predictors, filtering thresholds, or immunogenicity models. This can influence which antigens enter a vaccine. Computational methods, software versions, quality controls, and selection criteria should therefore be reported transparently. In personalized vaccine development, standardization is not a technical detail; it is part of biomarker reliability.

AI may improve the integration of genomic, expression, immunopeptidomic, single-cell, spatial, and clinical data, and may help define subgroups for shared vaccines or combinations. Its appropriate role remains prioritization. A high-ranking peptide is still a hypothesis until natural presentation, immune recognition, and the relevant tumor context are demonstrated.

## 8 Functional validation: From predicted antigen to therapeutic biomarker

The practical value of a vaccine biomarker ultimately depends on functional validation. A candidate antigen may be predicted from sequencing data, supported by RNA expression, and ranked favorably by HLA-binding algorithms, but these steps do not prove that the antigen is naturally presented or capable of inducing a useful immune response. For this reason, validation should be viewed as the point where a molecular candidate begins to become a therapeutic biomarker (Li et al., 2017).

Several levels of testing can contribute to this process. HLA-binding assays can confirm that a peptide interacts with the predicted restricting molecule, although binding alone is not sufficient. T-cell assays then address the next question: whether the antigen is recognized by functional lymphocytes. Enzyme-linked immunospot (ELISpot) assays, intracellular cytokine staining, activation-induced marker assays, tetramer or multimer staining, T-cell receptor sequencing, and cytotoxicity assays can each provide different information on the magnitude, phenotype, specificity, and function of the response (Macchia et al., 2013).

The most informative validation strategies are those that connect antigen recognition to tumor-cell control. T cells may respond to a synthetic peptide *in vitro*, but fail to recognize tumor cells if the peptide is not naturally processed, is presented at very low density, or is absent from the relevant tumor subclones. Co-culture systems using patient-derived tumor cells, organoids, or tumor slices can help bridge this gap by preserving some aspects of tumor biology and tissue context (Grönholm et al., 2021). These models are not perfect substitutes for clinical evidence, but they can test whether immune recognition remains meaningful in a more realistic tumor setting.

This point is especially relevant for personalized vaccines. Manufacturing time and tissue availability often limit the depth of validation that can be performed before treatment. In many cases, antigen selection must rely on a hierarchy of evidence rather than complete proof for every candidate. Still, even partial validation can improve target choice. A candidate supported by expression, HLA retention, immunopeptidomic evidence, and T-cell recognition is more convincing than a peptide selected only because it scores well computationally.

Functional validation also matters for shared neoantigen vaccines. In that setting, the same mutation may recur across patients, but presentation remains HLA-dependent and context-dependent. A shared mutant peptide is clinically useful only for patients whose tumors retain the appropriate HLA allele, express the mutant protein, process the relevant peptide, and present it at sufficient levels. This is why shared vaccines still require molecular and immunological stratification.

Preclinical models can add a final layer, especially when the goal is to test combinations or preventive/interception strategies. Humanized mouse models, syngeneic vaccination systems, and genetically engineered cancer models can help evaluate whether vaccination induces durable immunity, reduces tumor burden, or prevents tumor development in defined biological contexts (Yin et al., 2020). These models have limitations, particularly in reproducing human HLA diversity and tumor evolution, but they remain useful when interpreted cautiously.

Functional validation is therefore the evidential boundary between a candidate and a therapeutic biomarker. The strongest package links the alteration to natural presentation, immune recognition, and, when feasible, control of autologous tumor cells.

## 9 From vaccine platforms to clinical translation: an integrated framework

Translation begins after this evidence package has been assembled. The platform must induce the type and breadth of immunity required by the antigen; the clinical window must allow that response to develop; and any combination should address a demonstrated barrier. These choices are interdependent rather than consecutive add-ons.

Platform choice should therefore follow the biology of the target rather than technological enthusiasm alone. Peptide and long-peptide vaccines may be suitable for well-defined shared antigens, especially when HLA restriction and epitope presentation are reasonably understood. RNA and self-amplifying RNA platforms are well suited to personalized or multivalent antigen sets because they can encode several candidates within the same product. Dendritic-cell vaccines allow closer control over antigen loading and presentation, although they remain complex and individualized. Viral-vector, oncolytic, and *in situ* vaccination approaches may be useful when strong innate immune stimulation, local antigen release, or tumor inflammation is needed (Saxena et al., 2021; Lin et al., 2022). In this sense, platforms should not be viewed as interchangeable delivery systems. Each one is better matched to some antigen types, immune contexts, and clinical windows than to others.

The clinical window may be just as important as the platform. Therapeutic vaccines are often less convincing in bulky advanced disease, where tumor heterogeneity, immune suppression, stromal barriers, and antigen-loss variants are more established. By contrast, adjuvant treatment, minimal residual disease, and molecularly selected high-risk settings may offer more favorable conditions. In resected melanoma and pancreatic cancer, individualized neoantigen vaccines have provided encouraging evidence that vaccination may be more effective when tumor burden is low and immune monitoring can detect vaccine-induced T-cell responses (Weber et al., 2024; Rojas et al., 2023). In mismatch repair-deficient cancers and Lynch syndrome-related models, frameshift peptide vaccination also illustrates how recurrent neoantigens may support therapeutic or interception-oriented strategies (Kloor et al., 2020; Gebert et al., 2021). These examples do not prove that all vaccines should be restricted to early disease, but they suggest that timing should be considered a biological variable, not only a clinical convenience.

Combination therapy should follow the dominant mechanism of resistance. If a tumor already contains antigen-experienced T cells but these are restrained by PD-1/PD-L1 or CTLA-4 signaling, checkpoint blockade may help sustain vaccine-induced immunity (Pardoll, 2012). If the main barrier is stromal exclusion, TGF-β-rich fibrosis, abnormal vasculature, or myeloid suppression, other forms of microenvironmental modulation may be needed (Binnewies et al., 2018; Mariathasan et al., 2018; Tauriello et al., 2018; Fukumura et al., 2018). This is an important distinction. Combining a vaccine with another treatment should not simply mean adding an immune stimulant to a standard regimen. The combination should have a clear purpose: to improve priming, increase antigen presentation, support T-cell infiltration, reverse exhaustion, reduce suppressive myeloid activity, normalize vessels, or make the tumor tissue more accessible to effector cells.

Monitoring is also part of the therapeutic logic. A vaccine may induce circulating antigen-specific T cells, but this does not automatically mean that the tumor is being controlled. Useful monitoring should include several layers when feasible: expansion and persistence of antigen-specific T cells, changes in T-cell receptor clonality, cytokine or interferon-related signatures, evidence of tumor infiltration, antigen spreading, and molecular signs of residual disease. Persistent memory responses and epitope spreading after personalized neoantigen vaccination show that immune monitoring can capture effects that are not always visible through conventional radiological endpoints (Hu et al., 2021). Circulating tumor DNA may add complementary information by tracking molecular residual disease, recurrence, or clonal evolution over time (Abbosh et al., 2017). The most informative strategy will probably combine immune readouts with molecular evidence of tumor control.

Several translational barriers remain. Personalized vaccines require recent tumor material, high-quality sequencing, reliable HLA typing, rapid computational prioritization, manufacturing capacity, quality control, and coordination with the patient’s clinical course. Shared neoantigen vaccines may be easier to standardize, but they still require molecular selection and HLA compatibility. In both cases, computational pipelines must be transparent and reproducible, because different algorithms or filtering thresholds may select different antigens from the same tumor (Hundal et al., 2020; Xia et al., 2024). Cost, access, regulatory requirements, turnaround time, and trial design are therefore not secondary issues. They directly influence whether a biologically strong vaccine concept can become a practical treatment.

**FIGURE 3:**
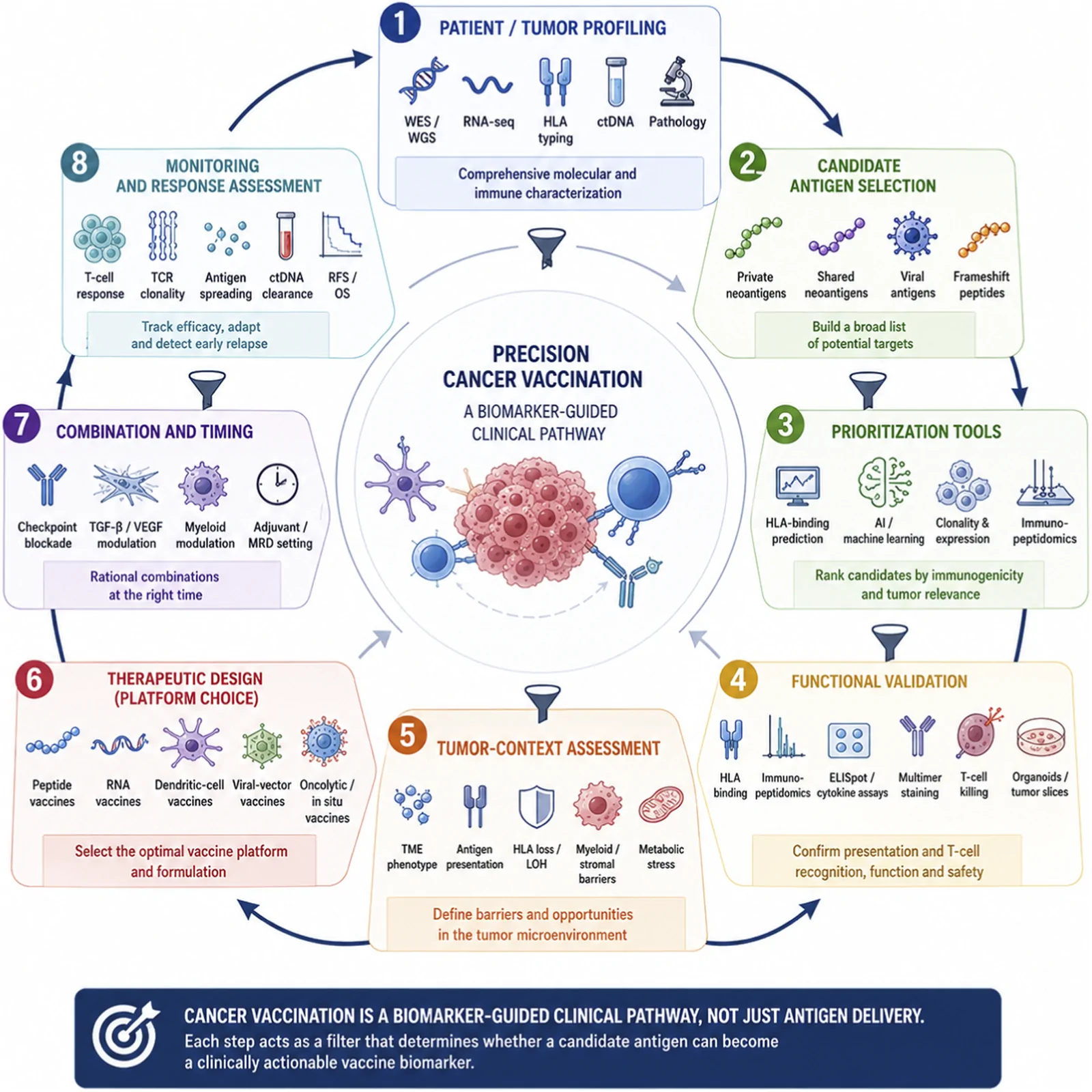
Integrated clinical decision framework for precision cancer vaccination. The dark outer arrows indicate the prospective sequence from patient/tumor profiling (step 1) to monitoring and response assessment (step 8). Funnel symbols mark the four explicit decision gates: prioritization (step 3), functional validation (step 4), tumor-context assessment (step 5), and combination/timing (step 7). At these gates, candidates may be excluded or the plan revised; steps 1, 2, 6, and eight are enabling or action stages rather than filters. The main forward path includes step 4 to step 5 and step 5 to step 6. The lighter inner arrows denote iteration: findings at step 5 may require renewed prioritization or validation, or may redirect platform choice, while monitoring can trigger repeat profiling. This conceptual figure was generated using ChatGPT (ChatGPT 5.5 Thinking) from author-defined scientific content and was subsequently reviewed, corrected, and finalized using GIMP 2.10.30.

Figure 3 translates these dependencies into a clinical workflow. It begins with tumor profiling and ends with longitudinal response assessment, while allowing feedback whenever prioritization, functional testing, or tumor context invalidates an earlier choice.

The framework is intended to exclude weak targets and poorly matched interventions early. Its value lies in making each additional assay, platform, or combination answer a defined biological question.

## 10 Conclusion

Cancer vaccines show why a molecular alteration is not automatically a therapeutic opportunity. The alteration must yield a naturally presented antigen, be recognized by functional lymphocytes, remain sufficiently distributed across the tumor, and operate in a tissue context where immune activity can matter. Preventive viral vaccines established the principle of cancer prevention by vaccination; neoantigen discovery, RNA platforms, immunopeptidomics, single-cell and spatial profiling, and improved monitoring have made therapeutic development more rational, but not simple.

That rationality carries a practical cost. A personalized product may require recent tumor and matched-normal material, genomic and transcriptomic sequencing, HLA typing, several computational pipelines, optional immunopeptidomics or functional assays, good manufacturing practice production, quality control, and serial immune and molecular monitoring. Each layer consumes tissue, time, specialist effort, and money, while technical depth does not guarantee clinical benefit (Saxena et al., 2021; Lin et al., 2022; Hundal et al., 2020; Xia et al., 2024). Development should therefore be proportionate: intensive personalization is defensible when the antigen set is credible, turnaround fits the clinical window, and the expected benefit exceeds simpler alternatives. Trials must connect immune readouts to recurrence, circulating tumor DNA clearance, progression-free or overall survival, toxicity, and patient-relevant outcomes rather than treating immunogenicity as an endpoint in itself.

The most credible opportunities remain molecularly selected patients, adjuvant or minimal-residual-disease settings, virus-associated cancers, hereditary high-risk states, and combinations directed at a defined resistance mechanism. Progress will depend less on declaring one platform superior than on showing that tumor biology, antigen validation, timing, delivery, and monitoring form a coherent therapeutic decision.
